# Hepatocyte Growth Factor Isoforms in Tissue Repair, Cancer, and Fibrotic Remodeling

**DOI:** 10.3390/biomedicines2040301

**Published:** 2014-11-05

**Authors:** Ognoon Mungunsukh, Elizabeth A. McCart, Regina M. Day

**Affiliations:** Department of Pharmacology, Uniformed Services University of the Health Sciences, Bethesda, MD 20814-4799, USA; E-Mails: ognoon.mungunsukh@usuhs.edu (O.M.); elizabeth.mccart@usuhs.edu (E.A.M.)

**Keywords:** hepatocyte growth factor, NK1, NK2, MET receptor, signal transduction, truncated isoforms, tissue repair

## Abstract

Hepatocyte growth factor (HGF), also known as scatter factor (SF), is a pleotropic factor required for normal organ development during embryogenesis. In the adult, basal expression of HGF maintains tissue homeostasis and is up-regulated in response to tissue injury. HGF expression is necessary for the proliferation, migration, and survival of epithelial and endothelial cells involved in tissue repair in a variety of organs, including heart, lung, kidney, liver, brain, and skin. The administration of full length HGF, either as a protein or using exogenous expression methodologies, increases tissue repair in animal models of tissue injury and increases angiogenesis. Full length HGF is comprised of an *N*-terminal hairpin turn, four kringle domains, and a serine protease-like domain. Several naturally occurring alternatively spliced isoforms of HGF were also identified. The NK1 variant contains the *N*-terminal hairpin and the first kringle domain, and the NK2 variant extends through the second kringle domain. These alternatively spliced forms of HGF activate the same receptor, MET, but they differ from the full length protein in their cellular activities and their biological functions. Here, we review the species-specific expression of the HGF isoforms, their regulation, the signal transduction pathways they activate, and their biological activities.

## 1. Introduction

Hepatocyte growth factor (HGF), or scatter factor (SF), was first identified as a factor from the plasma from humans and rabbits, and also rat platelets, that could induce the proliferation of hepatocytes in culture [1,2,3]. Following its initial discovery, HGF was demonstrated to be produced primarily by mesenchymal cell types, especially fibroblasts, in a variety of tissues including lung, heart, kidney, liver, skin, and brain [4,5,6,7,8,9,10,11,12,13,14]. HGF is required for normal embryonic development, and mice lacking HGF display failure for the development of multiple organs [9,15,16,17,18,19,20]. The HGF receptor, MET, was identified as a 145 kDa tyrosine kinase receptor with a single transmembrane domain, a juxtamembrane regulatory domain, and a *C*-terminal multifunctional docking domain that was phosphorylated in response to HGF [21,22]. Like HGF, MET is expressed in a wide variety of tissues [9,23,24,25,26,27,28]. However, in contrast with HGF, MET is localized primarily on epithelial and endothelial cell types [29,30,31]. Thus, in normal tissues, it is believed that HGF functions in homeostasis as a paracrine factor synthesized by mesenchymal cells to induce the survival and maintenance of the other cells of the tissue [9,26].

Shortly after the discovery of HGF, several isoforms of the factor were identified. Full length HGF was shown by Northern blots to correspond to an mRNA of about 6 Kb, encoding a protein of ~82,000 kDa, that is proteolytically processed to produce a ~69 kDa alpha subunit with a single disulfide bond to a ~34 kDa beta subunit [32]. Structurally, the full length HGF protein was shown to contain an *N*-terminal hairpin loop and four kringle domains in the alpha subunit, and a serine protease-like domain in the beta subunit [32,33]. An alternatively processed mRNA for human *HGF* was identified by Northern blot, with an estimated size of 1.5 kb, with a predicted translation product of ~33 kDa protein containing the *N*-terminal hairpin loop and the first two kringle domains of HGF (named NK2) [34,35]. A second splice variant of human HGF was later identified by Northern blotting, a ~1.2 kb transcript, encoding a protein of ~20 kDa protein containing the HGF *N*-terminal hairpin loop and the first kringle domain (named NK1) [36]. This later truncated isoform was also identified in murine mRNA [37]. Both the NK2 and NK1 isoforms were demonstrated to compete with HGF for binding to the same MET receptor [34,36,37,38,39]. However, the biological and cellular activities of the two truncated isoforms differ greatly from that of the full length HGF as does the regulation of their expression.

## 2. Hepatocyte Growth Factor (HGF) Isoforms

HGF is a high-molecular-weight polypeptide growth factors whose domain structure and mechanism of activation resemble those of the blood protease plasminogen, and belongs therefore to a family of plasminogen-related growth factors [33]. Besides HGF, this family includes the macrophage stimulating protein (MSP), an effector of macrophage chemotaxis and phagocytosis [40]. These two proteins share a common ancestral gene with the plasminogen and apolipoprotein. Phylogenetic studies suggest that these proteins evolved from a common ancestor that consisted of a single kringle domain and a serine protease (or serine protease-like) domain, separated by a region involved in their activation [33]. HGF and MSP have 45% sequence homology with each other, and 40% with plasminogen. Four main features distinguish HGF and MSP from plasminogen: (1) high-affinity binding to a specific membrane receptor, activating a complex cell-signaling pathway; (2) lack of enzymatic activity of the serine protease domain; (3) the number of kringle domains (four in HGF and MSP, five in plasminogen); (4) binding to heparan sulfate proteoglycans [20].

Functional orthologs of HGF and MET can be found in vertebrates from bony fishes to humans, and *HGF* and *MET* related gene sequences of limited length and without clear functional similarity can be found in invertebrates as well. The most common transcript variant of *HGF* is the full length *HGF*. The human orthologs are 4792 nt (XM_006715956), 2820 nt (NM_000601), and 2805 nt (NM_001010932) long transcripts that contain 18 exons [41]. The first two transcripts encode for a 728 aa polypeptide and last one encodes a 723 aa polypeptide.

The *NK1* isoform is the shortest functionally active isoform of *HGF*. In humans, it is transcribed as a 2079 nt-long transcript (NM_0010934) consisting of five exons that encode a 201 aa polypeptide. *NK1* sequences are predicted in several other species including the mouse (*Mus musculus*, NM_00128946), rabbit (*Oryctolagus cuniculus*, XM_008258180), opossum (*Monodelphis domestica*, XM_007504021), and non-human primates (e.g., *Chlorocebus sabaeus*, XM_007982358). They are highly similar among different species. Primates share ~98.5% sequence identity, and the murine and human *NK1* coding regions are 88.63% identical. The *NK1*-3'UTR of primates are ~95.7% identical, but the 3'UTR of rabbit and human share 71.57% identical sequence. The 3'UTR of the murine *NK1* is only 48.85% identical to the human. The *HGF* gene contains an alternative splice acceptor in the intron that follows the five coding exons among these species.

The other functionally active isoform of *HGF* is the *NK2*. It is transcribed in humans as a 1292 nt (NM_001010933) or 1307 nt (NM_001010931) mRNA of seven exons, encoding 284 aa or 290 aa, respectively.

**Figure 1:**
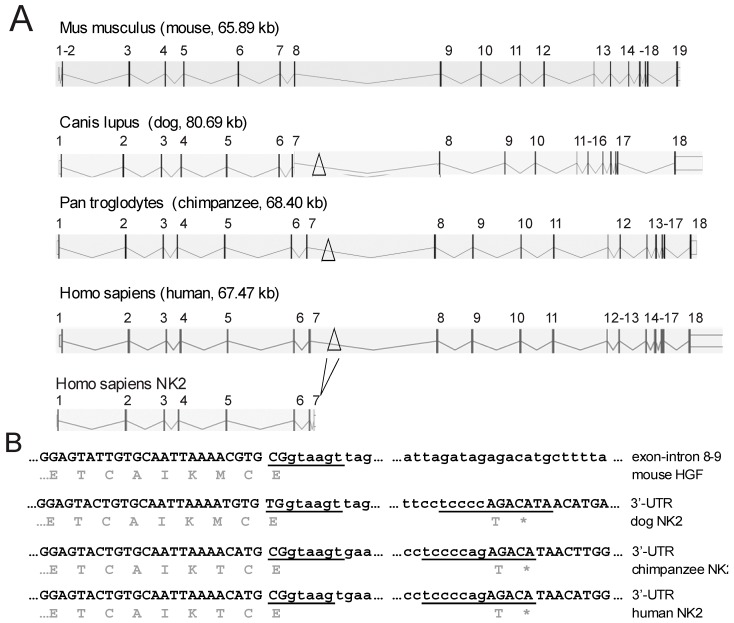
Generation of the *NK2* message. (**A**) A schematic comparison of hepatocyte growth factor (*HGF*) genes. Bars and numbers indicate exons. The alternative exon used for *NK2* splicing is indicated by a triangle at the corresponding introns. The murine intron does not contain this alternative exon; (**B**) The exon (capital letters)–intron (small letters) boundary sequences for *NK2* splicing. Predicted splice donor and acceptor sites are underlined. The murine sequence lacks characteristics commonly found in splice acceptor sites. The carboxy-terminal amino acids of *NK2* and the stop codon (*) are shown below the coding sequence.

**Figure 2:**
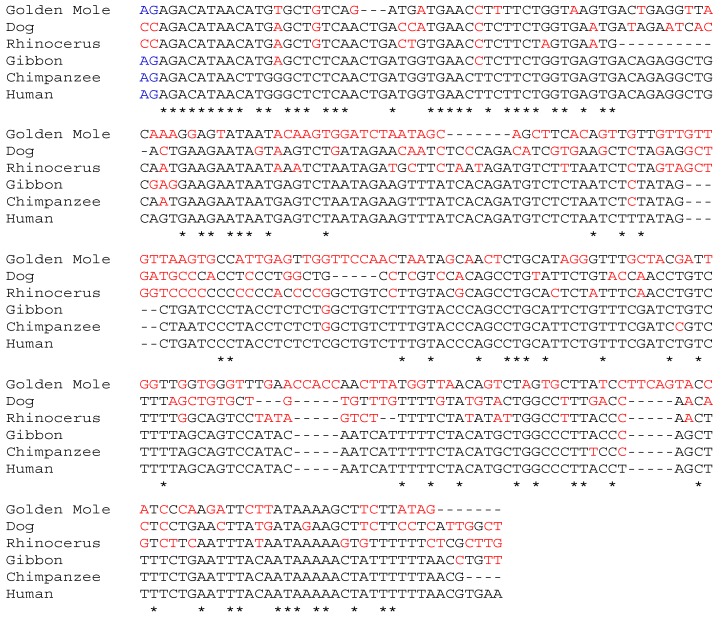
Multiple alignment of the human *NK2* 3'UTR with predicted 3'UTR sequences from species using the European Molecular Biology Laboratory—European Bioinformatics Institute Clustal Omega program [45]. Asterisks indicate nucleotides identical with the human sequence; non-identical nucleotides are shown as red letters. The first two nucleotides (AG, blue) are the predicted primate splice acceptor site.

The NCBI Gnomon [42] predicted *NK2* similar sequences in several other species like *Chrysochloris asiatica* (golden mole, XM_006834344.1), *Ceratotherium simum simum* (rhinoceros, XM_004431294) and *Canis lipus familiaris* (dog, XM_005630886), in addition to primates (Figure 1). Sequence analysis using Basic Local Alignment Search Tool (BLAST) [43] revealed that the murine *HGF* sequence does not contain the splice sites required for the generation of *NK2* (Figure 1) and data from our laboratory indicated that the *NK2* isoform was not expressed in mice [44]. Only primates share significant sequence similarities in the 3'UTR, e.g., *Pan troglodytes* (chimpanzee) XM_003318558 and *Nomascus leucogenys* (gibbon) XM_003252220 share 97% similarity with the two *NK2* isoforms (NM_001010933 and NM_001010931) of human (Figure 2). Although the sequences present in the NCBI Gnomon predict the expression of *NK2* in dog, rhinoceros, and golden mole, supporting experimental data have not yet been produced.

## 3. Regulation of Expression of *HGF* and Its Isoforms

*HGF* is expressed in most tissues, and both mRNA and protein have been detected in the liver, lung, kidney, skin, and brain. The *HGF* gene promoter has been extensively analyzed to determine the mechanism(s) of its regulation in development/growth, tissue repair, fibrosis, and oncogenesis [46,47,48]. *HGF* expression is positively regulated by other growth factors, such as epidermal growth factor, platelet-derived growth factor, and several members of the fibroblast growth factor family [49]. *HGF* expression is also increased in response to inflammatory cytokines interleukin (IL)-1α and -1β, IL-6, IL-8, and interferon-γ [50,51,52,53]; the regulation by these cytokines may be linked to *HGF* expression during early phases of inflammation that precede tissue repair. Analyses of the *HGF* promoter revealed functional *cis*-elements for transcription factor binding including IL-6 response elements, activating protein-2 (AP2) elements, nuclear factor-1 (NF1) elements, nuclear factor-IL6 elements, a cyclic AMP response element, several binding sites for the SP family of transcription factors, an upstream stimulatory factor (USF) element, a chick ovalbumin upstream promoter-transcription factor element, a complex C1 element, and the cell type specific CCAAT/enhancer binding protein (C/EBP) binding element [54,55,56,57]. Another element in the *HGF* promoter, the peroxisome proliferator-activated receptor gamma (PPAR γ)-responsive element, was shown to strongly regulate *HGF* expression in response to either PPAR γ 1 or PPAR γ 2 ligands, but because this binding site overlaps with the NF1 and chicken ovalbumin upstream promoter-transcription factor elements, the binding of these other factors repress PPAR γ activation [55]. Specific regulation of *HGF* in response to tissue injury and/or inflammation is believed to occur through factor binding to the C/EBP element [56].

*HGF* expression is tightly regulated, and several elements within the promoter suppress its expression. Binding of activating protein-2 (AP2) to a regulatory site −230 to −260 bp upstream of the transcriptional start site suppresses *HGF* expression [58]. Another repressor of *HGF* expression, a repeat of 30 deoxyadenosines (termed “deoxyadenosine tract element” or DATE) is located about 750 bp upstream of the *HGF* start site [59]. The DATE element is thought to be a hot spot for promoter mutations leading to *HGF* dysregulation in breast cancer [59].

*HGF* expression is potently suppressed in fibroblasts and other cell types by the pro-fibrotic cytokine transforming growth factor beta 1 (TGF-β1), by hypoxia, and by glucocorticoids [50,60]. In a human fetal lung fibroblast cell line, TGF-β1 down-regulated HGF protein and mRNA. Interestingly, the regulation of *HGF* occurred without affecting *NK2* expression in the cells [61]. Recent findings in human adult lung fibroblasts provide evidence that the selective regulation of *HGF* by TGF-β1 occurs through TGF-β1 up-regulation of miR-199a-3p [44]. The miR-199a-3p binds to the *HGF* mRNA 3'UTR leading to degradation of *HGF* mRNA. The *NK2* mRNA has an alternative 3'UTR sequence that is not regulated by miR-199a-3p [44].

## 4. Cellular Signaling by HGF and Its Truncated Isoforms

HGF is a pleiotrophic factor, inducing motogenesis, mitogenesis, survival, and in some cell types, morphogenesis [8,62,63]. HGF signaling occurs through its high affinity receptor, MET, a tyrosine kinase receptor [21,22]. Analysis of functional residues of the MET receptor revealed that, in addition to phosphorylation of tyrosines in the kinase domain (Y^1234^ and Y^1235^), two other phosphotyrosines in the carboxy-terminal multifunctional docking domain, Y^1349^ and Y^1356^, regulate downstream signaling [64,65,66,67,68]. Tyrosine phosphorylation of MET allows its interaction with adaptor proteins and signaling molecules, resulting in the downstream activation of major signaling pathways including Ras/p42/p44 mitogen activated protein kinase (MAPK), phosphatidylinositol 3-kinase (PI3K)/Akt, src kinase, protein kinase C isoforms (PKC), signal transducer and activator of transcription 3 (STAT3), small GTP-binding proteins Rap1, Rac, and Rho, and [69,70,71,72,73,74,75,76,77]. A number of investigations have attempted to identify individual signaling pathways required for the various biological effects of HGF, and there appears to be substantial overlap [76,77,78,79]. While mitogenic and antiapoptotic actions of HGF appear to require p42/p44 MAPK, PI3K/Akt, and STAT3, the motogenic activity requires PI3K and src kinase [76,77,78,79]. Morphogenesis and tubulogenesis appear to require PI3K as well as other pathways downstream of the Grb2 adaptor protein [79]. HGF inhibition of apoptosis from a variety of causes including oxidative stress, DNA damaging agents, or signaling by angiotensin II has been shown to require PI3K/Akt, p42/p44 MAPK, and STAT3 [78,80,81,82,83,84].

Following their initial discoveries, it was demonstrated that NK1 and NK2 bound to MET and induced its phosphorylation in a competitive manner with full length HGF [34,36,38,85]. In early studies, it appeared that the level of total MET tyrosine phosphorylation by the truncated isoforms in cell cultures of breast epithelial cells was somewhat reduced compared to MET phosphorylation by full length HGF [38]. However, later studies with higher amounts of refolded, purified protein, the MET phosphorylation could be induced by the truncated isoforms to the same total levels as with full length HGF [36,85,86].

NK1 induced motility in Madin Darby canine kidney (MDCK) cells and 32D cells expressing MET [85,87,88,89]. At high concentrations, NK1 was found to induce proliferation in a breast cancer epithelial cell line and in BALB/MK cells, MCF-10A cells, and in BaF3 cells expressing MET, albeit at a lower level than HGF [36,85,87,90,91,92]. However, NK1 was not shown to induce proliferation in the A549 lung cancer cell line, possibly due to lower levels of the factor that were used [38]. High concentrations of NK1 were also reported to be capable of inducing branching morphogenesis in endothelial cells [87]. In BALB/MK cells, NK1 was demonstrated to activate p42/p44 MAPK at levels similar to full length HGF [89]. In a non-tumorigenic human prostate epithelial cell line, NK1 was demonstrated to activate p42/p44 MAPK, Akt, srk kinase, p125 focal adhesion kinase, SMAD2/3, and STAT3 [93]. Although NK1 has been demonstrated to recapitulate most of the actions of full length HGF, it is not known whether the signaling of NK1 in cells is the same as for full length HGF.

Like NK1, NK2 induced motility in MDCK cells, but NK2 failed to induce motility in human umbilical vein endothelial cells [85,86,87]. In contrast, NK2 did not induce proliferation in any cell line tested, and NK2 did not induced branching morphogenesis [34,85,86,87]. Furthermore, NK2 could competitively inhibit HGF-induced proliferation and morphogenesis [85,87]. Interestingly, examination of NK2-induced signal transduction in cell culture demonstrated that NK2 was capable of activating both PI3K and p42/p44 MAPK in 32D cells expressing MET and in a breast cancer epithelial cell line, suggesting that these two pathways were not sufficient to induce proliferation in response to MET activation [86]. In another effort to characterize NK2-induced signaling *in vivo*, a study of melanoma cells in NK2 transgenic mice demonstrated that the tissues of these mice had reduced MET phosphorylation and reduced p42/p44 MAPK activation in contrast with HGF transgenic mice, suggesting that signaling by this isoform *in vivo* is compromised [94]. The signal transduction by NK2 in normal non-transformed cells remains poorly understood.

## 5. Biological Functions of HGF and Its Isoforms

### 5.1. HGF Isoforms during Development

HGF plays an essential role during development in the placenta [95], liver [15], and kidney [17,96]. HGF is also critical for normal neuronal development and for limb skeletal muscle [16,97,98,99]. Consequently, knockout mice for *HGF* die *in utero* during early stages of embryogenesis [99]. A pattern of HGF secretion, along with other essential growth factors, has been noted in a variety of developmental epithelial layer, including in the intestinal lamina propria, where epithelial and mesenchymal interactions are essential for normal epithelial cell differentiation [100,101].

Although the expression patterns and effects of HGF have been investigated in development, the roles of the two naturally occurring isoforms of HGF, NK1 and NK2, in development are not yet understood. One study of HGF isoform expression during development in macaques found low levels of expression of both NK1 and NK2 as well as novel forms of NK1 and NK2 (dNK1 and dNK2) with a five amino acid deletion in the first kringle domain [102]. Increased HGF and all isoforms were found to be increased at 144 days of gestation, and all forms were found in the endometrium and placenta [102]. The role of the expression of the HGF isoforms during embryogenesis and development is not yet known.

### 5.2. Role of HGF Isoforms for Tissue Homeostasis and Repair

HGF is a growth factor, survival factor, and mitogen for epithelial and endothelial cells from the lung, skin, kidney, liver, heart, and brain [103,104,105,106,107,108,109,110,111]. In the adult, basal expression of HGF is believed to be important for normal tissue homeostasis, where the local production of HGF by resident mesenchymal cells maintains the specialized epithelium of the tissue [9,10,13,26,28,29,63,112,113,114,115,116,117]. For instance, in the kidney, expression of HGF by mesangial cells in the tissue microenvironment is believed to maintain normal growth of kidney endothelial cells [112], and HGF is required for trans-telencephalic migration of interneurons and for neuronal survival for brain cell homeostasis [118].

HGF expression is greatly increased in response to most types of tissue injury [5]. HGF expression is induced in animals after experimental renal, cardiac, pulmonary, adrenal gland, or hepatic injury [1,112,115,119,120,121,122,123,124,125,126,127,128]. For the vascular endothelium, HGF is a potent proliferative factor critical for angiogenesis, a process often required for repair of tissue injury [129]. Interestingly, the lung was also shown to synthesize HGF in response to injuries in distal organs, suggesting a paracrine function for tissue repair by the lung [130]. Increased expression of HGF in response to tissue injuries was demonstrated to be related to tissue repair activities *in vivo* [131,132]. Additionally, the time course of HGF up-regulation or administration of HGF correlated with the increased proliferation of epithelial cells following experimentally-induced tissue injury [119,133,134]. Direct evidence for the role of HGF in tissue repair was provided in experiments in which the blockade of HGF using neutralizing antibodies resulted in the inhibition of hepatocyte proliferation for liver regeneration and kidney regeneration following injury [135,136]. Also, the generation of a conditional knockout of HGF in mice was demonstrated to result in impaired liver regeneration following CCl_4_ treatment [137]. Severely impaired liver generation was also observed after partial hepatectomy in a conditional MET mutant mouse with MET-null phenotype [138]. The failure in normal liver repair was characterized by altered cell cycle progression and cell cycle entry. Others have shown that conditional knockout of MET caused hypersensitivity to FAS-induced apoptosis of hepatocytes and impaired recovery from centrolobular lesions as a result of persistent inflammatory reaction, overproduction of osteopontin, early calcification, and suppressed hepatocyte migration into the injured area [139]. Wound healing experiments with mice in which MET was conditionally inactivated in the epidermis demonstrated that MET-deficient mice required twice as much time as wild type mice for the healing and only MET positive cells that escaped knockout recombination were involved in this delayed skin healing [140].

The administration of HGF, whether as a purified protein or through ectopic expression methods, was demonstrated to increase normal tissue repair in experimental injury models of the lung, skin, liver, kidney, heart, pancreas, and brain, and improves angiogenesis [24,31,131,141,142,143,144,145,146,147,148,149,150,151,152,153,154,155,156,157]. Of critical importance, HGF administration increases normal repair processes and inhibits fibrotic remodeling and/or scar tissue formation [24,142,145,148,150,156,158,159,160,161,162,163,164,165,166,167]. Currently, non-viral plasmids for the expression of HGF are being explored for efficacy in treatment of critical limb ischemia and for peripheral neuropathy in diabetes [168,169].

The basal expression of NK1 and NK2 has been observed in multiple tissues, including in normal fibroblasts from the lung [44]. Interestingly, the expression of NK1 and NK2, but not full length HGF, was observed in chondrocytes [170]. In general, the normal biological function of the truncated isoforms is not known. Over-expression of the HGF truncated isoforms *in vivo* has been shown to have varying effects on cellular proliferation, survival and migration, cellular events that are all believed to be required for normal tissue repair.

In cell culture, NK1 has been shown to act as a mitogen and anti-apoptotic factor for normal hepatocytes in culture [171]. Transgenic mice over-expressing NK1 had similarities to mice over-expressing full length HGF, including “enlarged livers, ectopic skeletal-muscle formation, progressive renal disease, aberrant pigment cell localization, precocious mammary lobuloalveolar development” [37]. However, in NK1 transgenic mice, some of these abnormalities were reduced compared with HGF transgenic mice. For instance, in NK1 mice, livers were enlarged 1.5-fold, compared with 2–3-fold increases observed in HGF mice, and incidences of cancers were lower in the NK1 mice [37]. In another study, NK1 over-expression was observed to increase hepatocyte proliferation *in vivo* and to reduce liver fibrosis in murine partial hepatectomy model [171]. Administration of recombinant NK1 protein was also demonstrated to induce the proliferation of isolated islets in culture and to induce proliferation pancreatic β-cells in a murine model of type 2 diabetes [172].

Murine models of tissue injury suggest that NK2 does not induce the proliferation of normal cells and may inhibit normal tissue repair. NK2 transgenic mice did not exhibit the phenotypic consequences observed in response to HGF over-expression, including the inhibition of hyperplastic lesions of the kidney or olfactory mucosa, and abnormalities of the mammary gland and skeletal muscle [94]. NK2 transgenic mice also did not display gastrointestinal obstruction, progressive renal disease, or enlarged livers [94]. Interestingly, metastasis of malignant melanoma cells were extremely activated in NK2 transgenic mice compared to the wild type. The number of metastatic cells in the liver were nearly the same as that obtained with HGF transgenic mice, and the size of metastatic tumors in the NK2 livers were equivalent to wild type [94]. Additionally, NK2 over-expression in combination with HGF over-expression in bi-transgenic mice blocked the HGF-induced alterations [94]. At the cellular level, NK2 was demonstrated to inhibit HGF-induced proliferation of normal hepatocytes in culture and *in vivo* [94,147]. In a murine model of liver repair after partial hepatectomy, it was found that NK2 over-expression blocked liver tissue regeneration [173].

In an early investigation of a murine model of liver injury, the inducible expression of HGF both prevented the induction of liver injury and induced tissue repair [147]. In contrast, this report demonstrated that NK2 augmented CCl_4_-induced liver injury, but did not inhibit endogenous hepatocyte proliferation following liver injury [147]. In this study, the final level of liver recovery was not examined [147]. In a later study of NK2 over-expression following CCl_4_-induced liver injury in mice, it was found that NK2 promoted liver fibrosis and prevented normal tissue repair [174].

### 5.3. HGF Isoforms in Cancer

Expression of HGF and/or its receptor MET is increased in a wide variety of cancers [175]. The HGF/MET axis is believed to contribute significantly to proliferation, metastasis, extracellular matrix remodeling, tumor cell survival, and angiogenesis associated with tumors [176,177,178]. *MET*, originally identified as a proto-oncogene [179,180], was found to be up-regulated in a variety of cancers, inducing its constitutive expression [181,182,183,184,185,186]. Oncogenic forms of MET have been detected that induce constitutive activity and in some cases new functions [179,187,188]. HGF over-expression has also been demonstrated to be sufficient for neoplastic transformation in cells expressing normal levels of wild type MET, and HGF over-expression in transgenic mice drives the development of multiple cancers [189,190,191,192,193,194]. Because of the widespread expression of MET and HGF in cancer, their expression has been analyzed for cancer severity, and the degrees of expression of MET and HGF are believed to indicate the progression of disease as well as serve as prognostic markers for some forms of cancer [195,196,197,198].

Early studies suggested that NK1 and NK2 did not induce cancer cell proliferation in cell culture, and that the activation of motility was also reduced in some cancer cell types. For instance, both NK1 and NK2 inhibited the proliferation and motility of A549 lung cancer cell line *in vitro* [176]. In these same cells, both NK1 and NK2 altered the expression of matrix metaloproteinases, suggesting that the mechanism for the reduction of cellular motility in part lay in altered interactions with the extracellular matrix and increasing the adhesion capacity of the cells [176]. However, later studies suggested that NK1 at higher concentrations could induce both mitogenic and motogenic activities in a variety of cancer cells [36,199]; these activities were demonstrated to be improved in the presence of cell surface heparin or other mechanisms to induce NK1 dimerization [88,90,200]. In contrast, NK2 failed to induce proliferation of most cancer cells, and had varying effects on cancer cell motility [94,201].

*In vivo* studies challenged the original views of NK1 and NK2 as inhibitors of cancer. Transgenic mice over-expressing NK1 had a number of similar oncogenic events as found in mice over-expressing full length HGF, including mammary, hepatocellular, and melanocytic tumors [37]. In transgenic mice over-expressing NK2, NK2 inhibited HGF-induced melanoma cell proliferation *in vivo* [94]. *NK2* gene transfer was also demonstrated to inhibit proliferation of glioma cells *in vivo* [201]. However, in the melanoma model system, NK2 over-expression did induce higher levels of metastasis of the cancer cells [94].

## 6. Clinical Applications of HGF Isoforms

HGF has been used successfully as both protein and DNA as a therapeutic agent in preclinical animal models for ischemic heart disease, renal fibrosis, pulmonary fibrosis, and for other diseases where there is a need for increased tissues repair [142,148,163,164,202,203]. In humans, HGF has been investigated for the clinical treatment of myocardial injury (NIH clinical trial identifier: NCT01233336). To date, these studies have revealed that HGF is an early marker of myocardial injury and prognostic factor for post myocardial infarction. Other clinical trials (NCT00189540 and NCT00060892) in critical limb ischemia revealed that *HGF* gene therapy is safe and improved wound healing, reduced the necessity for amputation, improved pain at rest, and improved hemodynamic measurement without adverse effects on the quality of life in the critical limb ischemia population. HGF is also under investigation for treatment of diabetic peripheral neuropathy [169]. Furthermore, plasmid expression of *HGF* is in Phase III Clinical Trials to treat severe peripheral arterial disease in Japan [204], and adenovirus mediated *HGF* gene-transfer is under investigation as a potential treatment for coronary artery disease in a Phase I Clinical Trial in China [205]. Phase I/II Clinical Trials of recombinant human HGF protein are currently underway for the treatment of acute spinal cord injury (NCT02193334).

NK4, an artificial HGF isomer, contains the *N*-terminal hairpin domain and the subsequent four-kringle domains, and has been shown to act as an antagonist to HGF monogenic activity [206]. Based on the abundant preclinical data appears to be a potential role for the use of *NK4* in gene therapy for the treatment of cancer, including pancreatic cancer, gastric carcinoma, hepatocellular carcinoma, breast and endometrial cancer, lung cancer, and prostate cancer [207,208,209,210,211,212,213,214]. Due to its activity in the suppression of inflammatory cytokine production by CD4^+^ T cells, preclinical models have also shown that NK4 may provide a new approach for the treatment of rheumatoid arthritis [215,216]. The use of NK4 in ovarian cancer is currently under investigation.

In contrast with full length HGF and the synthetic HGF antagonist NK4, the naturally occurring isoforms of HGF are not currently in clinical trials. Although NK1 has been shown to induce all the signal transduction pathways of full length HGF, its lower level activity suggests that it may not be as beneficial as full length HGF for clinical applications. Additionally, NK2, though its ability to antagonize HGF proliferation could be useful for anti-cancer therapy, however its partial activation of MET may also preclude this application.

## 7. Conclusions

The naturally occurring isoforms of HGF, NK1 and NK2 are expressed in human tissues during development and in normal adults. Studies in cell culture and in transgenic animals suggest that NK1 is capable of recapitulating normal HGF signaling and biological activities, while NK2 appears to be an antagonist for HGF-induced cellular proliferation. NK2 expression is increased relative to full length HGF in human fibrotic organ diseases, and it is possible that NK2 may play a role in the failure of normal repair. The normal biological roles of the HGF truncated isoforms remain to be determined. Further understanding of the normal functions of these proteins may provide insight for their use in human diseases.
